# Analysis of the effect of hashimoto's thyroiditis and insulin resistance on ovarian volume in patients with polycystic ovary syndrome

**DOI:** 10.1186/s12905-023-02200-x

**Published:** 2023-02-24

**Authors:** Gülcan Gencer, Aliye Nigar Serin, Kerem Gencer

**Affiliations:** 1 Department of Biostatistics and Medical Informatic, Faculty of Medicine, Afyonkarahisar Health Sciences University, Afyonkarahisar, Turkey; 2 Department of Gynecology and Obstetrics, Faculty of Medicine, Karamanoglu Mehmetbey University, Karaman, Turkey; 3 Distance Education Application and Research Center, Afyonkarahisar Health Sciences University, Afyonkarahisar, Turkey

**Keywords:** PCOS, Insulin resistance, Ovarian volume, Hashimoto thyroid

## Abstract

**Objective:**

In this study, patients were divided into two groups. Patients with polycystic ovary syndrome (PCOS) and patients with polycystic ovary syndrome + Hashimoto's Thyroid (PCOS + HT). The effect of insulin resistance on ovarian volume in patients divided into two groups and the change in ovarian volume with the addition of HT to PCOS will be investigated.

**Material and methods:**

46 PCOS patients and 46 PCOS patients diagnosed with HT were included in this study. A detailed medical history was taken from all participants. Polycystic ovary image was evaluated as below or above 10 ml and antral follicles were counted by transvaginal ultrasound. Insulin resistance of the patients was evaluated according to the fasting insulin (HOMA) index.

**Results:**

Insulin resistance was found to be associated with fasting insulin, HOMA index, body mass index and right ovarian volume in patients diagnosed with PCOS. Among the patients diagnosed with PCOS + HT, insulin resistance was found to be significantly correlated with fasting insulin, HOMA index, (BMI), (SHBG) and left ovarian volume. An increase in right ovarian volume was found in 37.5% of patients with PCOS without insulin resistance and in 76.3% of patients with insulin resistance. An increase in left ovarian volume was found in 35.7% of patients without insulin resistance diagnosed with PCOS + HT and in 68.8% of patients with insulin resistance.

**Conclusions:**

This study shows that ovarian volume should be evaluated in every PCOS patient in order to predict insulin resistance, which causes long-term metabolic diseases, and that all PCOS patients with increased ovarian volume should be investigated for insulin resistance. In addition, it has been observed that insulin resistance affects left ovarian volume in patients with PCOS + HT, whereas insulin resistance affects the volume of the right ovary more in patients with PCOS. At least one ovary has been found to be affected by long-term metabolic diseases. While there was a greater increase in ovarian volume with the addition of insulin resistance, no significant change was observed in the number of patients with increased ovarian volume (PCOS-58, PCOS + HT-57) with the addition of HT finding.

## Introduction

Polycystic ovary syndrome (PCOS) is the most common hyperandrogenic disorder [1]. Although various genetic and environmental theories have been proposed for the biological (as well as pathological) changes that occur during the course of the disease, the Hyperandrogenism theory and the insulin resistance theory are widely accepted. Inflammatory and autoimmune causes have also been reported due to its close relationship with insulin resistance and thyroid disorders [2]. PCOS affects many body functions [3]. PCOS leads to infertility and early miscarriage [4, 5]. The main endocrine disorders responsible for clinical manifestations are hyperandrogenemia and abnormal insulin response to glucose [5, 6]. It has been shown that hypothyroidism causes many metabolic disorders such as a decrease in glucose excretion or uptake by muscles or adipose tissues in response to insulin, an increase in sex hormone binding globulin, weight gain and hyperlipidemia [4, 6]. Giampaolino et al. [7] prepared a review summarizing the available literature. First, although an association with Hyperandrogenism has been identified, it is still unclear, second, its role in chronic low-grade inflammation by activating the immune system with increased production of proinflammatory cytokines interfering with insulin receptor function causing IR (Insulin Resistance)/hyperinsulinemia, as well as its gastrointestinal role. Hormones such as ghrelin and peptide YY (PYY), bile acids, interleukin-22 and Bacteroides vulgatus are highlighted [7].

## Materials and methods

A total of 92 patients, 46 PCOS patients aged between 18–35 years, and 46 patients with both PCOS and HT, who applied to the Antalya Training and Research Hospital, Obstetrics and Gynecology outpatient clinic and endocrinology and metabolic diseases polyclinic were included in the study. Written and verbal informed consent was obtained from the patients for the study.PCOS was diagnosed in the presence of at least two of the Rotterdam criteria. Both married and patients between the ages of 18–35 were included in the study. Possible etiological causes (congenital adrenal hyperplasia, androgen-secreting tumor, Cushing's syndrome) were excluded. Patients with ovarian pathology such as endometrioma, dermoid or simple cyst or who had ovarian surgery were excluded from the study group. All women with hypothyroidism were excluded from both groups. Being fertile was defined as a woman who had never had a problem conceiving.

### Clinical measurements

Group [8]. Oligoanovulation was clinically determined by its presence. The presence of hirsutism, acne or alopecia was taken as the clinical determinant of hyperandrogenism. The hirsutism scores of the patients were determined using the modified Ferriman-Gallwey (mFG) scoring system. With this method, hair density was scored between 0 and 4 in a total of nine anatomical regions: upper lip, chin, chest region, back, waist, lower and upper abdomen, upper arms and legs. Those with a total score of 8 and above were considered hairy. In ultrasonography, they were divided into two groups as > 10 cm^3^ below and above. Ultrasonographic evaluation of the ovaries was performed transvaginally in the lithotomy position with a MINDRAY brand DC-7 T model ultrasonography device. Patients with ovarian pathology such as endometrioma, dermoid or simple cyst or who had ovarian surgery were not included in the study group. All women with hypothyroidism were excluded from both groups. Evaluation of the patients included in the study was started with history and physical examination. In the first application of all cases, height (m) and weight (kg) were measured and BMI was calculated as kg/m2. Waist circumference was measured at the level of the navel, and hip circumference was measured at the level of the wide trochanter. In the early follicular phase of the menstrual cycle (days 2–5), 5 ml blood sample was taken and Low Follicle stimulating hormone (FSH), luteinizing hormone (LH), 17-hydroxyprogesterone (17-HOP), SHGB, free T3, free thyroxine (In Beckman Coulter DXI 800 device, FT4), fasting insulin, testosterone, DHEA-S levels were studied with Beckman Coulter commercial kits using chemiluminescence method. The following formula was used to determine insulin resistance: fasting plasma insulin (mIU/L) x fasting plasma glucose (mmol/L)/22.5. Values of 2.5 and above were accepted as insulin resistance.

### Statistical analysis

Analyzes were made with R. 4.2.2 package program and Phyton 3.7.12 program. While evaluating the findings obtained in the study, Fisher's Exact test or Pearson chi-square test was used to analyze the relationships between categorical variables. *P* values less than 0.05 were considered statistically significant. A fully connected feed-forward artificial neural network Multi Layer Perceptron (MLP) was used to classify patients as patients with PCOS or patients with PCOS + HT by volume count analysis of the right and left ovaries.

## Results

When the patients with and without insulin resistance were compared among the 46 PCOS patients included in the study, it was found to be associated with fasting blood glucose, BMI, HOMA index and right ovarian volume (Table 1). Variables that differed were found to have significantly higher values than those without insulin resistance. When the patients diagnosed with PCOS + HT were compared with and without insulin resistance, a significant correlation was found between HOMA index, fasting blood glucose, BMI, SHBG and left ovarian volume (*p* < 0.05) (Table 2). were found to be significantly higher than those without insulin resistance. SHBG, on the other hand, had a higher value than those without insulin resistance.

**Table 1:** Comparison of patients with polycystic ovary syndrome with and without insulin resistance

Variables	Without insulin resistance N = 8	With insulin resistance N = 38	*p* value
**PCOS**			
HOMA index	1.32 ± 0.8	6.2 ± 10.03	*p* < 0.05*
Age	28.25 ± 2.81	26.05 ± 3.95	*p* > 0.05
Fasting Blood Sugar	7.87 ± 6.83	27.76 ± 43.14	*p* < 0.05*
BMI	24.01 ± 3.71	27.42 ± 5.56	*p* < 0.05*
DHEA-S	356.77 ± 111.92	339.55 ± 135.81	*p* > 0.05
T	0.73 ± 0.48	0.88 ± 0.5081	*p* > 0.05
SHBG	26.35 ± 12.45	27.03 ± 18.32	*p* > 0.05
FSH	5.91 ± 1.52	6.35 ± 1.81	*p* > 0.05
LH	11.2 ± 6.76	11.15 ± 7.3	*p* > 0.05
Right Ovarian Volume	0.37 ± 0.51	0.76 ± 0.43	*p* < 0.05*
Left Ovarian Volume	0.50 ± 0.53	0.57 ± 0.50	*p* > 0.05

PCOS: Polycystic ovary syndrome HOMA: Homeostatic Model Assessment, BMI: Body mass index; DHEA-S: Dehydroepiandrostenodionesulfate, T: Total testosterone; SHBG: Sex hormone binding globulin; FSH: Follicle stimulating hormone; LH: Luteinizing hormone, *Statistically significant

**Table 2:** Comparison of patients with polycystic ovary syndrome and Hashimoto's Thyroid with and without insulin resistance

Variables	Without insulin resistance N = 8	With insulin resistance N = 38	*p* value
**PCOS + HT**			
HOMA index	1.36 ± 0.5	6.06 ± 7.53	*p* < 0.05*
Age	28.14 ± 3.37	27.62 ± 3.19	*p* > 0.05
Fasting Blood Sugar	10.78 ± 7.21	28.53 ± 36.66	*p* < 0.05*
BMI	24.78 ± 4.37	28.16 ± 4.62	*p* < 0.05*
DHEA-S	294.62 ± 133.42	311.97 ± 146.69	*p* > 0.05
T	0.88 ± 0.68	0.97 ± 0.52	*p* > 0.05
SHBG	58.08 ± 53.25	29.07 ± 17.2	*p* < 0.05*
FSH	8.69 ± 8.20	6.10 ± 2.06	*p* > 0.05
LH	7.88 ± 8.23	7.34 ± 4.03	*p* > 0.05
Right Ovarian Volume	0.57 ± 0.51	0.68 ± 0.47	*p* > 0.05
Left Ovarian Volume	0.35 ± 0.49	0.68 ± 0.47	*p* < 0.05*

PCOS: Polycystic ovary syndrome HT: Hashimoto’s Thyroid, HOMA: Homeostatic Model Assessment, BMI: Body mass index; DHEA-S: Dehydroepiandrostenodionesulfate, T: Total testosterone; SHBG: Sex hormone binding globulin; FSH: Follicle stimulating hormone; LH:Luteinizing hormone, *Statistically significant

For patients with PCOS, the difference in ovarian volume in patients with and without insulin resistance was examined (Table 3). Right ovarian volume was below 10 cm^3^ and within normal limits in 62.5% of patients without insulin resistance and 23.7% of patients with insulin resistance. In 37.5% of patients without insulin resistance and 76.3% of patients with insulin resistance, right ovarian volume was above 10 cm^3^ and ovarian volume was increased. There was a significant difference in left ovarian volume between patients with and without insulin resistance (*p* < 0.05). Left ovarian volume was less than 10 cm^3^ and within normal limits in 50% of patients without insulin resistance and in 42.1% of patients with insulin resistance. In 50% of the patients without insulin resistance and in 57.9% of the patients with insulin resistance, the left ovarian volume was above 10 cm^3^ and the ovarian volume was increased. There was no significant difference in left ovarian volume between patients with and without insulin resistance (*p* > 0.05) (Table 3).

**Table 3:** Comparison of patients with polycystic ovary syndrome with and without insulin resistance in terms of ovarian volume

	Without Insulin Resistance	With Insulin Resistance	Total	*p* value
**PCOS**				
Right ovarian volume < 10	5	9	14	*p* < 0.05*
	62.50%	23.70%	30.40%	
Right ovarian volume > 10	3	29	32	
	37.50%	76.30%	69.60%	
Left ovarian volume < 10	4	16	20	*p* > 0.05
	50.00%	42.10%	43.50%	
Left ovarian volume > 10	4	22	26	
	50.00%	57.90%	56.50%	
Total	8	38	46	

PCOS: Polycystic ovary syndrome, *Statistically significant

For patients with PCOS + HT, the difference in ovarian volume in patients with and without insulin resistance was examined (Table 4). Right ovarian volume was below 10 cm^3^ and within normal limits in 42.9% of patients without insulin resistance and in 31.3% of patients with insulin resistance. Right ovarian volume was greater than 10 cm^3^ and ovarian volume increased in 57.1% of patients without insulin resistance and in 68.8% of patients with insulin resistance. There was no significant difference in right ovarian volume between patients with and without insulin resistance (*p* > 0.05). Left ovarian volume was below 10 cm^3^ and within normal limits in 64.3% of patients without insulin resistance and 31.3% of patients with insulin resistance. Left ovarian volume was greater than 10 cm^3^ and ovarian volume increased in 35.7% of patients without insulin resistance and in 68.8% of patients with insulin resistance (Table 4). There was a significant difference in left ovarian volume between patients with and without insulin resistance (*p* < 0.05).

**Table 4:** Comparison of patients with polycystic ovary syndrome and Hashimoto's Thyroid with and without insulin resistance in terms of ovarian volume

	Without insulin resistance	With insulin resistance	Total	*p* value
**PCOS + HT**				
Right ovarian volume < 10	6	10	16	*p* > 0.05
	42.90%	31.30%	34.80%	
Right ovarian volume > 10	8	22	30	
	57.10%	68.80%	65.20%	
Left ovarian volume < 10	9	10	19	*p* < 0.05*
	64.30%	31.30%	41.30%	
Left ovarian volume > 10	5	22	27	
	35.70%	68.80%	58.70%	

PCOS: Polycystic ovary syndrome, HT: Hashimoto’s Thyroid, *Statistically significant

A fully connected feed-forward artificial neural network Multi Layer Perceptron (MLP) was used to classify patients as patients with PCOS or patients with PCOS + HT by volume count analysis of the right and left ovaries. In Table 5, a fully connected feedforward artificial neural network classified patients with PCOS and PCOS + HT with an accuracy rate of 53.2609%. There were actually 46 PCOS and 46 PCOS + HT patients in the study, but the differential diagnosis of PCOS or PCOS + HT could not be made with a high accuracy rate due to ovarian volume increase. Because, as seen in Tables 3 and 4, there was no significant change in the number of patients with increased ovarian volume with the addition of HT and increase in volume in only one ovary in two separate patient groups (PCOS-58, (PCOS + HT)-57).

**Table 5:** Classification of patients using right and left ovarian volumes

Methods	Confusion matrix	
	PCOS + HT	PCOS
Multi layer perceptron	9	37
	6	40

PCOS: Polycystic ovary syndrome, HT: Hashimoto’s Thyroid

Table 6 explores the effect of taking combined thyroid replacement therapy on excess volume. In Table 6, the effect of whether the patient received thyroid replacement therapy or not on the ovaries is not statistically significant (*p* > 0,05).

**Table 6:** The relationship between thyroid hormone replacement therapy and ovarian volume

PCOS + HT	Thyroid hormone replacement therapy		Total	*p* value
	No	Yes		
Right ovarian volume < 10	6	10		0.756
	30%	38.50%	34.8%	
Right ovarian volüme > 10	14	16		
	70%	61.50%	65.2%	
Left ovarian volume < 10	6	13	19	0.232
	30%	50%	41.3%	
Left ovarian volüme > 10	14	13	27	
	70%	50%	58.7%	

PCOS: Polycystic ovary syndrome, HT: Hashimoto’s Thyroid

## Discussion

Polycystic ovary syndrome (PCOS) is a complex and heterogeneous endocrine disease characterized by clinical or laboratory hyperandrogenism, oligo-anovulation and metabolic abnormalities, including insulin resistance, overweight or obesity, type II diabetes, dyslipidemia, and an increased risk of cardiovascular disease [9]. The most important clinical manifestation of PCOS is hyperandrogenism.

About 50–70% of these patients are hyperinsulinemic insulin resistant and suffer from metabolic syndrome, which alone increases the risk of type II diabetes and cardiovascular disease. Great importance has been attached to the relationship between insulin resistance and hypothyroidism for decades, and extensive studies have been conducted on these issues.

Moghetti [10] stated that it is estimated that approximately 70% of women with PCOS are insulin resistant, but this figure is affected by frequent referral bias. He also suggested that hyperinsulinemia plays a role in increased androgen overproduction, with bidirectional links between insulin resistance and hyperandrogenism.

Diamanti-Kandarakis et al. [11] emphasized that insulin resistance is an important feature that worsens the features of PCOS in women with PCOS.

Roos et al. [12] observed an interesting increase in the level of insulin resistance parameters even below very small decreases in thyroid hormone levels (59T1259T). These findings brought to mind the question of whether the intensity of hypothyroidism has an additional effect on insulin resistance, so they investigated whether HT accompanying PCOS would cause an additional ovarian increase in this situation.

Shorakae et al. [13] included forty-nine women with PCOS and 23 control groups in the analysis. showed that muscle sympathetic nerve activity (MSNA) and testosterone level, age and BMI were most significantly associated with PCOS status.

[14] found a significant relationship between BMI, HOMA, fasting blood glucose, and fasting glucose/insulin ratio (G/I ratio) between PCOS patients with and without insulin resistance. In addition, HOMA has been used to examine insulin resistance among PCOS patients of different ethnicities and in many articles dealing with insulin resistance in diabetics.

SHBG is a well-known marker of insulin resistance in diabetics [15] and low levels have been reported in adolescent girls (known to be at risk for PCOS and insulin resistance) [16]. It has been shown that lower SHBG levels are associated with lower G/I ratios and higher HOMA indices (consistent with insulin resistance) in women with PCOS [17].

Thyroid disorders, especially hypothyroidism, are more common in patients with PCOS. Subclinical hypothyroidism may exacerbate insulin resistance in PCOS patients [3].

Legro et al. [14] conducted a comparative study on insulin secretion, insulin resistance, and thyroid function in patients with polycystic ovarian syndrome with and without Hashimoto's thyroiditis. Patients with PCOS and HT had higher insulin secretion and IR levels, while free thyroxine and thyrotropin levels were found to be significantly lower than those without HT. found that the ratio of free thyroxine to thyrotropin was higher in HT patients. It has been shown that in patients with PCOS, HT may be associated with insulin resistance and relatively low thyroid function.

Enzevaei et al. [3] included 19 (25.5%) of 75 PCOS patients with subclinical hypothyroidism and 56 (74.4%) euthyroid patients. The prevalence of insulin resistance was 22.7%, and it was found that 77.3% of the patients had no insulin resistance and were normal. showed that they could not find a relationship between insulin resistance and subclinical hypothyroidism in PCOS patients.

Adams et al. [18] characterized the appearance of more than 10 follicles 2–8 mm in size with enlarged ovaries and pearl necklace-like peripherally located due to the increase in stroma tissue by USG as PCOS. For this reason, the relationship between enlarged ovaries and PCOS, which is characterized by insulin resistance, was investigated. There are various studies on this subject.

Wakimoto et al. [19] evaluated the relationships between ovarian volume and AMH, LH/FSH ratio, T concentrations and body mass index (BMI). While there was a significant relationship between BMI and ovarian volume and BMI and T, other hormone parameters did not find a significant relationship. There was a significant difference between the patients diagnosed with PCOS and the control group in terms of HOMA index, mean ovarian volume, fasting blood glucose, and FAI [20]. In the literature, there are other score deductions according to the ovarian volume to be taken as a reference [21]. An ovarian volume of less than 10 ml was considered normal, and a volume of 10 ml or more was considered as increased ovarian volume.

The metabolic changes observed in HT and PCOS are heterogeneous; however, they are often related. They usually encompass higher BMI, glucose and lipid abnormalities. Metabolic disorders in Hashimoto's disease are more pronounced in patients with overt or subclinical hypothyroidism than in euthyroid patients receiving T4 replacement therapy [22]. PCOS is often associated with high prevalence of hyperinsulinemia, insulin resistance, and obesity. These patients are at increased risk for metabolic syndrome, type 2 diabetes mellitus (T2DM), cardiovascular disease, and unopposed estrogenic effects on the endometrium [23]. The LH/FSH ratio value is very dependent on the assay used to measure hormones. These patients should be evaluated for the risk of developing the metabolic syndrome and its components, such as T2DM, hypertension, and hyperlipidemia. Overweight women with PCOS may have an increased risk of acute myocardial infarction and stroke [24]. It has many common features in the pathogenesis of obesity and PCOS. Both disorders are linked to insulin resistance and hyperinsulinemia [25].

Many studies show the relationship between insulin resistance and hyperandrogenemia. In our study, volume increase was detected in at least one ovary of patients with high insulin resistance for patients with PCOS and patients with PCOS + HT. In addition, the addition of HT did not exacerbate this increase, but the addition of insulin resistance exacerbated this increase and doubled it.

## Conclusion

It is unclear whether PCOS has significant effects on insulin resistance parameters due to the diversity and variability of findings from various studies. This difference in insulin resistance parameters may be due to ethnic diversity among patients. On the other hand, this study shows that ovarian volume should be evaluated in every PCOS patient and all PCOS patients with increased ovarian volume should be investigated for insulin resistance in order to predict insulin resistance that causes long-term metabolic diseases. In addition, it has been observed that insulin resistance affects left ovarian volume in patients with PCOS + HT, while insulin resistance affects the volume of the right ovary more in patients with PCOS. In addition, it has been determined that long-term metabolic diseases may affect at least one ovary.

When the finding of insulin resistance is also added to patients with PCOS, it was observed that the volume of the right ovary increased more than 2 times, while the volume of the left ovary increased slightly. When the finding of insulin resistance was also added to patients with PCOS + HT, the opposite situation was encountered. This time, it was observed that the volume of the left ovary increased approximately 2 times, while the volume of the right ovary increased slightly. Due to the limitations of the study not being able to increase the number of patients, more patients are needed to say that the left ovarian volume of patients with PCOS increases. It is recommended to be confirmed by increasing the number of patients in subsequent publications. There were also 46 PCOS and 46 PCOS + HT patients in the study, but the differential diagnosis of PCOS or PCOS + HT could not be made with high accuracy due to the increase in ovarian volume.

## Data Availability

The datasets used and/or analysed during the current study available from the corresponding author on reasonable request.
